# Wearable Devices, Smartphones, and Interpretable Artificial Intelligence in Combating COVID-19

**DOI:** 10.3390/s21248424

**Published:** 2021-12-17

**Authors:** Haytham Hijazi, Manar Abu Talib, Ahmad Hasasneh, Ali Bou Nassif, Nafisa Ahmed, Qassim Nasir

**Affiliations:** 1 Department of Informatics Engineering, CISUC-Centre for Informatics and Systems of the University of Coimbra, University of Coimbra, P-3030-790 Coimbra, Portugal; haytham@dei.uc.pt; 2 Intelligent Systems Department, Palestine Ahliya University, Bethlehem P-150-199, Palestine; 3 College of Computing and Informatics, University of Sharjah, Sharjah P-27272, United Arab Emirates; anassif@sharjah.ac.ae (A.B.N.); nafisa.ahmed@sharjah.ac.ae (N.A.); nasir@sharjah.ac.ae (Q.N.); 4 Department of Natural, Engineering, and Technology Sciences, Arab American University, Ramallah P-600-699, Palestine; Ahmad.Hasasneh@aaup.edu

**Keywords:** artificial intelligence, decision fusion, COVID-19 detection, heart rate variability, natural language processing, wearables

## Abstract

Physiological measures, such as heart rate variability (HRV) and beats per minute (BPM), can be powerful health indicators of respiratory infections. HRV and BPM can be acquired through widely available wrist-worn biometric wearables and smartphones. Successive abnormal changes in these indicators could potentially be an early sign of respiratory infections such as COVID-19. Thus, wearables and smartphones should play a significant role in combating COVID-19 through the early detection supported by other contextual data and artificial intelligence (AI) techniques. In this paper, we investigate the role of the heart measurements (i.e., HRV and BPM) collected from wearables and smartphones in demonstrating early onsets of the inflammatory response to the COVID-19. The AI framework consists of two blocks: an interpretable prediction model to classify the HRV measurements status (as normal or affected by inflammation) and a recurrent neural network (RNN) to analyze users’ daily status (i.e., textual logs in a mobile application). Both classification decisions are integrated to generate the final decision as either “potentially COVID-19 infected” or “no evident signs of infection”. We used a publicly available dataset, which comprises 186 patients with more than 3200 HRV readings and numerous user textual logs. The first evaluation of the approach showed an accuracy of 83.34 ± 1.68% with 0.91, 0.88, 0.89 precision, recall, and F1-Score, respectively, in predicting the infection two days before the onset of the symptoms supported by a model interpretation using the local interpretable model-agnostic explanations (LIME).

## 1. Introduction

SARS-COV-2 (COVID-19) was first reported in Wuhan, China, at the end of 2019 [1] and then spread across China and many countries globally in a few months, leading to a continuous pandemic throughout the world. As of March 2020, the World Health Organization (WHO) [2] has declared that this virus is a global epidemic and is spreading exponentially, as the number of infected people up to the date of preparing this research paper has exceeded 260 million cases. More than 5 million have died in more than 200 different countries worldwide [3].

The ability to quickly identify, monitor, and isolate COVID-19 patients is one of the most significant challenges that persist even after nearly two years following the first announcement of the virus. Thus, the early detection of COVID-19 is predominant to minimize the widespread of the infection, particularly for asymptotic patients, and take responsible isolation measures.

Currently, the viral nucleic acid amplification test (e.g., polymerase chain reaction, known as PCR) is the primary diagnosis technique worldwide [4]. However, due to the limited capacity of laboratories, test kits, and health care units, and this test’s cost, early detection techniques of this disease became necessary. That is why, since December 2019, numerous artificial intelligence (AI) techniques have already been proposed and developed to detect and classify this virus’s inflammatory signs, mainly using computed tomography (CT) and X-ray images [5,6,7], and recently using physiological signals such as in [8,9,10].

The CT imaging techniques helped in detecting the virus. However, due to the high cost and the risk of catching the virus from those devices themselves, these techniques would seem less practical.

Therefore, this work presents a framework that utilizes physiological signals acquired from biofeedback wearable devices (e.g., smartwatches) and smartphones in the presymptomatic screening of the COVID-19 using different AI techniques to predict the diseases before the onset of the symptoms.

Wearable, cheap biofeedback devices that are widely used and commercially available became a potential tool to detect the early onsets of the infection. Wearable devices such as smartwatches allow continuous measurements of physiological signals such as heart rate variability (HRV), skin temperature, resting heart rate, respiratory rate, oxygen saturation, perspiration, and ambulatory ‘subjects’ activity 24/7 [8]. Papaioannou et al. showed an established association between HRV and inflammatory markers. The HRV shows an inverse relationship with nonspecific inflammatory marker C-Reactive Protein (CRP) [11]. CRP level can be a prognostic indicator in COVID-19 patients [12]. Recently, some studies indicated that higher levels of CRP are associated with pneumonia infection [13]. Moreover, Karjalainen et al. [14] showed that a one °C increase in body temperature could increase heart rate by 8.5 beats per minute (BPM) on average.

Therefore, wearables could be utilized to detect the early signs of the COVID-19 inflammatory. However, those signals are not highly sensitive nor specific to the COVID-19 inflammatory manifestation, and thus many supporting techniques, including AI models, would help characterize the COVID-19 infection patterns.

We are aware that biometric sensors (and wearable devices) would not be an alternative to the standardized screening biochemical tests, such as PCR or antigen detection. Instead, these biometric solutions are supposed to augment the screening/detection methods using affordable and commercially available wearable devices and smartphones that individuals use prevalently.

In this paper, we consider daily HRV and BPM changes in addition to daily ‘volunteers’ survey responses as the primary sources of information to different classification models. The goal is to investigate the capacity of AI models to discriminate between healthy physiological signals (i.e., HRV and BMP) and affected physiological signals due to the COVID-19 infection. The characterization of COVID-19 inflammatory patterns in physiological signals would enable the development of alerting mobile applications that aggregate HRV data from wearables with the support of ‘users’ daily input statuses to early identify abnormal HRV behavior and link it to early signs of COVID-19 infection passively. Since physiological signals, including the HRV measures, are nonspecific for the COVID-19 nor other viral inflammatory onsets, ‘users’ tweets (statuses) that express their daily feelings would optimize the decision and minimize the false-positive rates and the false-negative rates. Alerting users before the onset of symptoms would enable them to take preventive measures such as self-isolation or undertake the authorized COVID-19 tests.

The automated decision from the model can subsequently be relayed to physicians to allow timely intervention. Wearable biosensing technology, which is low-cost and commercially available, can play an essential role in reducing the overwhelmed health centers and hospitals. The advantages of wearables over other supporting techniques can be represented by two main points: (a) the low cost and the availability of biosensors in contrast to the imaging techniques, which are considered of a high cost; (b) the possibility to diagnose, monitor, and control remotely without any human interaction at the early stages. Nonetheless, AI techniques are immensely required to guarantee a reliable decision about the infection.

In this paper, we use AI in two folds: the classification of HRV signals into binary classes (i.e., potential infection or no sufficient evidence of disease) and the natural language processing of user input status using the fastai text classification library [15]. The two models generate prediction probabilities for each class, which are linearly combined to produce the final decision reliably.

To the best of our knowledge, this is the first work that utilized HRV features to detect potential signs of COVID-19 supported by the local interpretable model-agnostic explanations (LIME) to explain the AI decision. Moreover, the integration of user context (i.e., health daily status) represented by the daily textual logs (provided by users) with the biometric data (acquired from wearables and smartphones) to support the final decision (potentially infected or no sufficient evidence) is an aspect of this work’s novelty.

The rest of the paper is organized as follows. The related work on COVID-19 detection techniques is discussed in Section 2, while Section 3 describes the proposed methodology, the participants, and the dataset. Section 4 presents and discusses applying the AI model to biometric and non-biometric data. Section 5 discusses the threats to validity. Finally, Section 6 presents the conclusion and the future work.

## 2. Related Work

Although most of the proposed artificial intelligence (AI) approaches to detect, diagnose, or monitor COVID-19 patients have focused on using image-based techniques, some other approaches have addressed this problem as a sensor-based task. Image-based techniques have mainly relied on CT images and X-ray images [5,6,7,16,17,18,19,20,21,22,23]. However, since a limited number of COVID-19 X-ray or CT-scan images are currently available, training and building an image-based model might therefore be difficult and challenging at this time. Even if a model has been built using the available data, the model cannot be generalized due to the limited number of images used to train the model. Therefore, investigating this problem as a sensor-based task would be more convenient, where several recent sensor-based works have also achieved promising results.

Wearable biosensor and smartwatch technologies have recently emerged. They can be used to measure a combination of physiological data, such as heart rate, heart rate variability, respiration rate, and other vital signals. In a recent review that appeared in the “Sensors” journal [17], Channa et al. indicated that the use of wearables has grown during the COVID-19 pandemic and still growing. In this review [17], the authors explained the importance of wearable’s contributions to prevent the pandemic. Grzesiak, et al. in [18] show that using wearable devices in physiologic monitoring may detect impending viral infection before the development of symptoms. Some wearable devices or ‘biosensors’ vital signals have already been used to detect COVID-19 positive individuals [19,20,21]. As COVID-19 is closely associated with respiratory rate changes, some recent research has focused on breath analysis to detect coronavirus disease. For instance, Giovannini et al. discussed the technical aspects of applying breath analysis in diagnosing COVID-19 [22]. The authors expect that breath-based detection methods would significantly reduce unnecessary exposure to contagious bodies and help contain pandemic’s spread. The work in [22] discussed using a multiplexed nanomaterial-based sensor array for detecting COVID-19 in exhaled breath. With the evolving studies in biosensors and their role in combating COVID-19, simpler sensing methods were introduced, such as the study of Fedorin et al. [23]. The authors chose to use consumer smartwatches for respiratory events screening. The use of commercial wearables has the advantage of affordability and availability in markets. However, due to their relatively low precision, they could not characterize whether the detected events are related to COVID-19 or other similar viral diseases.

Nonetheless, with the support of predictive AI models, biosensing wearable studies in combating COVID-19 have emerged for detecting the diseases before the symptoms of COVID-19 manifest. For example, Dean J Miller et al. [24] proposed a model trained through gradient boosted classifiers to identify COVID-19 individuals two days before symptoms appeared, based on changes detected in the respiration rate. Their model only identified 20% of the asymptotic COVID-19 individuals, while 80% were identified on the third day after the symptoms appeared. In a more recent study, Quer et al. [25] developed a mobile application to collect smartwatch and activity tracker data in addition to self-reported responses and testing results from individuals to predict COVID-19 infection in symptomatic patients. Similarly, Ates et al. [26] showed that wearable devices could be used in the early detection of COVID-19 in asymptomatic and presymptomatic cases. These studies used heart rate, number of daily steps, and sleep time as predictors of the infection.

Other studies additionally included temperature data in their models besides the other physiological measured data (e.g., HR, HRV, RR, etc.) [9,27,28]. In [28], the authors used other biosensors besides the smartwatch, such as a pulse oximeter, to measure systolic blood pressure as an additional source of information for the COVID-19 detection. Another example is shown in the study of M. U. Ashraf et al. [29]. The authors proposed an IoT framework that uses both wearable sensors for measuring temperature and pulse rate as well as non-wearable for measuring respiratory rate and blood pressure where the latter is attached to the entrances of public places. This framework aims to detect positive COVID-19 individuals, identify the suspected human to human infection, and monitor the suspected individuals.

On the other hand, some recent attempts have proposed to build a combination of physiological data and cough sounds model to detect COVID-19 [30,31].

HRV has witnessed significant attention in predicting COVID-19 symptoms manifest and acute responses among all the heart-related metrics. Hasty et al. [12] showed that a substantial decrease in HRV preceded CRP elevations, indicating acute inflammation response with a positive predictive value of 90.9%. However, in these kinds of studies, it is challenging to control correlating daily HRV and CRP levels in patients. Nevertheless, HRV proved to be a good predictor of the infection in the early stages before the onset of symptoms. For example, in [32], ZI Khalpey et al. show that HRV can be used for early detection and prediction of COVID-19, which helps physicians intervene earlier and reduce spread and mortality. Likewise, Khalpey et al. [10] show that HRV standard deviation of the interbeat interval of normal sinus beats known as SDNN differed between individuals in two periods: seven days before and seven days after COVID-19. However, this study was based on statistical methods used in analyzing the patterns of the HRV and did not use any AI techniques to optimize the prediction. In [33], Ponomarev et al. support the claim of using HRV at the individual level as a predictor of the COVID-19 infection. The authors in [33] show that HRV shows a statistically significant change among individuals during the COVID-19 infection period.

One can see that most of the literature models focus on analyzing biosensing signals with other subjective assessments to detect COVID-19 infections in individuals using AI techniques. To the best of our knowledge, no studies have addressed combining HRV time domain and frequency domain features and user health textual status to be used to detect COVID-19. Moreover, we noticed a lack of studies that give attention to the explainability of the AI model. Therefore, finding the optimal combination of physiological and subjective data (i.e., textual logs of users) supported by explainable AI remains an open question and needs further research investigation. This paper proposes a different AI technique and a decision fusion model that uses a set of physiological features and daily ‘users’ input status to predict the potential signs of infection in a two-day time window before the onset of the infection. The main contributions in this research study are as follows:

- Different prediction techniques were proposed, namely two independent prediction models: one for the HRV measures and the other is for analyzing the daily textual status of users as reported by them using NLP techniques.
- A model interpretation based on the LIME framework was introduced to better understand each feature’s contribution to the final decision.
- An accuracy of 83.34 ± 1.68% with 0.91, 0.88, and 0.89 precision, recall, and F-score, respectively, were obtained in predicting the infection two days before the onset of the symptoms.
- The decision fusion technique between the biometric model decision and the non-biometric model decision (i.e., feelings and reported status) improved the accuracy and the precision of the obtained results.

## 3. Participants, Dataset, and Methods

This work aims to investigate the capacity of AI techniques to classify the biomarkers extracted from wearable devices and classify natural language generated by users about their daily status to predict COVID-19 infection. The following subsections provide an overview of the participants, the dataset used in this study, and the methodology to analyze, classify, and predict the onset of COVID-19 infection.

### 3.1. Participants

**Figure 1:**
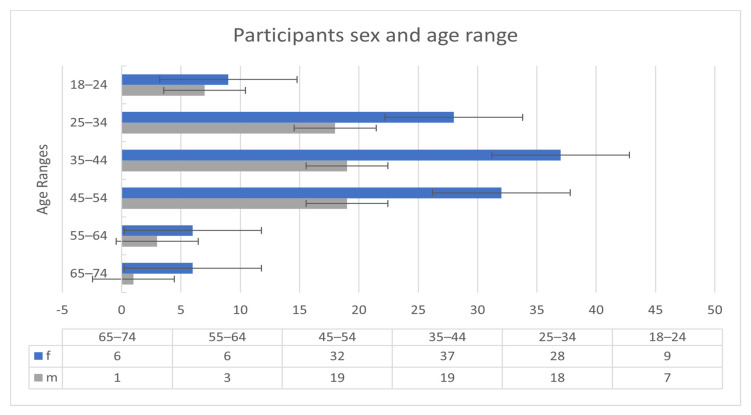
Participants demographic information.

The participants of the Welltory study [34] consisted of 186 patients. The 186 participants were all COVID-19 infected. Nonetheless, measurements and readings before and after the infection are available for some patients. Figure 1 shows the sex and age ranges of the participants.

**Figure 2:**
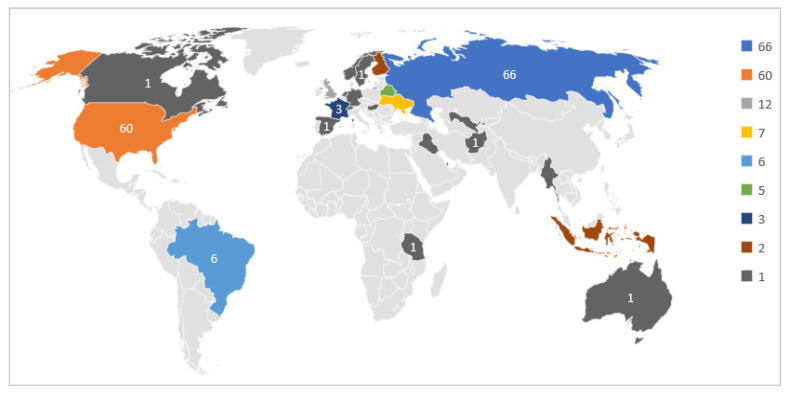
Participants Geographical Distribution.

According to the Welltory study, those participants consented to the use of their data for research purposes. Although the number of participants is limited, the diversity of the participating countries is interesting to study further, as shown in Figure 2.

As we can notice, most participants are from Russia and United States. The participants used different wearables, such as Fitbit, Garmin, and Apple watches. Besides, they used the Welltory mobile application to collect HRV data and textual responses to daily status questions. The following section explains the data collected from the wearables and the mobile application.

### 3.2. Dataset Description

The current work is based on a publicly available dataset [33] that Welltory provided within a research work. Their research work was focused on monitoring physiological changes of COVID-19 patients. The physiological signals and other vita signals were collected through wearables, e.g., Fitbit, Apple Watch, and Garmin, that were integrated with a mobile application to enable patients to log their daily status as text tags (e.g., what feelings are experienced). Although all participants were COVID-19 infected, some patients could share their previous (healthy) HRV data with the application. Therefore, labeling the available data in CSV format as either healthy or infected for training purposes was possible. The participants in the Welltory study shared their heart-related measurements through photoplethysmography (PPG) technology. PPG is an optical method that can be used for heart rate monitoring by assessing changes in blood volume under the skin. PPG is available in wearables like smartwatches and mobile phones through optical sensors. The interest in assessing HRV through analyzing PPG (called pulse rate variability or PRV). The PRV is calculated through the estimation of time intervals between consecutive peaks in the PPG signal [35]. Various studies (e.g., [35,36]) showed that PRV features can be used as a surrogate for HRV indexes (usually extracted from ECG signal). The dataset does include not only HRV data only, but also several vital signs, as shown in Table 1 along with textual data. The textual data resulted from daily participants logs, such as short answers about their status or COVID-19 test results. The onset of the symptom’s dates is provided for 79.8% of the participants, where 20.2% did not provide an onset date and this is either because they were asymptomatic patients, or they did not provide the date.

As observed from Table 1, different attributes can be significant markers to infer an early infection of respiratory viral infection and potentially Coronavirus infection with the support of other attributes and readings.

To detect abnormalities in the physiological patterns of the patients, the readings should have a reference baseline. Thus continuous readings at least several days before the onset of symptoms and after the recovery are necessary. Unfortunately, the current dataset lacks this property to some extent. The current dataset included 186 subjects that were reported as COVID-19 patients. Less than 60% had consistent readings and a relatively established baseline in their healthy time (over two weeks). Despite the physiological measures regarding intra- and inter-variability among subjects, one of the potential solutions for dealing with imbalanced datasets in the next work is to train the model from established datasets of healthy people, including heart-related measures. Successful examples of applying transfer learning or using pre-trained models in chest x-ray images data for predicting COVID-19 can be seen in [37,38].

Regarding the various wearable brands that were used in the study, it is critical to understand their accuracy and measurement errors which might significantly affect the decision-making in later stages. As mentioned in this section, HRV (or PRV) measurements were acquired optically through PPG sensors embedded in commercial wearables and smartphones. Some studies (e.g., [39]) demonstrate that PPG signal potential inaccuracies might stem from: (a) diverse skin type; (b) motion artifacts; (c) signal crossover. In [39], the authors show no statistically significant difference in the accuracy of wearables (i.e., Apple, Fitbit, and Garmin) across skin types. However, they found significant differences between devices across activity types (e.g., rest, physical movement). They concluded that, at rest, Apple Watch had the lowest mean absolute error compared to the true measurement from an ECG.

### 3.3. Methods

**Figure 3:**
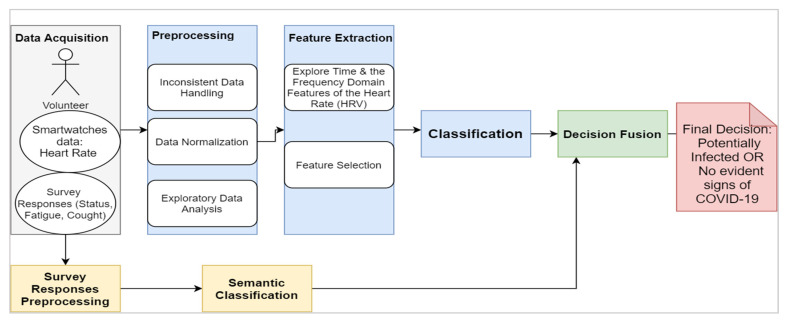
Schematic Representation of the Approach.

The proposed framework, as shown in Figure 3, consists of four main blocks:

1. The data acquisition: The physiological signals (i.e., HRV) were collected from participants through the Welltory application over a continuous period. The collected data included textual logs as a part of ‘participants’ daily reports on the application. The text’s tags comprised short words like tired, fever, fatigue, back to active life, and other short messages.
2. The preprocessing: This step included cleaning the data that do not conform with the required standards, such as having data before and after the onset, having correct onset dates, and having sufficient daily logs. The second step was to normalize the data due to the variable nature of HRV among participants by using the direct max-min normalization as appears in the following formula:
  (1) $$x\;\mathrm{normalized}=\frac{x-\min\left(x\right)}{\max\left(x\right)-\min\left(x\right)}\;\mathrm{where}\;x\;\mathrm{is}\;\mathrm{HRV}\;\mathrm{feature}$$
3. Exploratory data analysis (EDA): This aims to visualize and test the data distributions and patterns before introducing them to the AI models.
4. Feature extraction and selection: In this stage, domain knowledge and data-driven approaches are utilized. In the domain knowledge, we selected the HRV measures among other vital signs mentioned in Table 1 due to many reasons: (a) the well-established connections in the literature between HRV features and pathological changes including inflammatory onsets [40,41], (b) the timely manner response of some HRV features such as the standard deviation of NN intervals (SDNN) and the root mean square of successive differences between normal heartbeats (RMSSD) [42]. Nonetheless, HRV is still nonspecific to certain inflammatory infections like the COVID-19. Thus, we fused the model with non-physiological complementary data like the textual information tweeted by participants. From the initial screening of the textual tags posted on the application, we noticed a recurrent pattern of words expressed among those who started to feel unwell due to the COVID-19 infection (before the actual onset of symptoms). This additional source of information would be useless with asymptomatic patients as they would not report any significant feelings. Thus, HRV features remain the primary source in our work. Examples of HRV time-domain and frequency-domain are listed below:
  (a) The time-domain features:
    - Beat per minute (BPM).
    - Meanrr: The mean between two RR intervals.
    - Mxdmnn: The difference between the maximum and minimum RR intervals.
    - SDNN: The standard deviation of all the normal-to-normal RR intervals.
    - RMSDD: The root mean square of successive differences between each heartbeat.
    - pNN50: The mean number of times the changes in the normal-to-normal intervals exceed 50 ms.
  (b) The frequency-domain features:
    - HF: The high frequency of the heart rate represents the activity in the 0.15–0.40 Hz range.
    - LF: The low frequency of the heart rate represents the activity in the 0.04–0.15 Hz range.
    - LF/HF: The ratio between the low and high frequencies.

We focused our attention on the HRV features and the daily textual logs. Although the number of HRV time domain and frequency domain features is limited, we used data-driven filter approaches to select the best features based on the analysis of variance (ANOVA) [43] and mutual information [44]. ANOVA and Mutual Information feature selection are convenient with the numerical input data and categorical target data.

The selected HRV features from the filter-based feature selection methods are then fed into a set of state-of-the-art classifiers, namely support vector machine (SVM), K-nearest neighbor (KNN), and logistics regression (LR) using leave-one-out cross-validation (LOOCV). The selection of LOOCV is performed to avoid training bias for the same subject. In LOOCV, each observation is considered a validation set, and the N-1 is the training set.

Regarding the natural language processing of the daily logs, we used the fastai library [15] to preprocess, analyze the texts and classify them based on their content. We used language_model_learner function and text_classifier_learner based on pretrained model. The text learner is based on recurrent neural network(RNN) long short-term memory (LSTM) used for sequence learning tasks, including semantic classification. The model initializes a pretrained weight dropped LSTM with a drop probability of weight of 30%. The original model was pretrained on wikitext (28,595 preprocessed Wikipedia articles and 103 million words) [45]. Then, we fine-tuned this pretrained model on our data which consisted of textual tags from the study participants expressing their daily feelings. Our team labeled the data file according to ground-truth information about the onset of symptoms or test results date. To ensure the model does not forget what it learned previously, the fastai technique uses gradual unfreezing (GU). GU includes freezing all pre-trained weights, and after each epoch, it unfreezes a layer, from the last one to the first.

The resulting classification probabilities from both classifiers (i.e., the HRV and the Text classifiers) are combined using the weighted linear combination as a decision fusion approach as appears in the following formula:

(2) final P=α∗P1+(α−1)∗P2

where P1 and P2 correspond to the prediction probability of the first and the second classifiers, respectively. α is the weight of each classifier. The best α = 0.7 for the HRV classifier from empiric results and 0.3 for the language classifier. Although the HRV measure is nonspecific for the COVID-19 nor the inflammatory responses in general, it is still more indexing to the onset of COVD-19 infection than the ‘participants’ expressions about their daily physiological and psychological statuses, and this explains the weights used in Formula (2) above.

## 4. Results and Discussion

The dataset provided the HRV features over time for COVID-19 patients (some of them before and after the onset of the COVID-19 symptoms). The dataset offered other information gathered through wearable smartwatches and a mobile application. This paper investigated a part of the whole dataset, namely the HRV measurements and the ‘participants’ daily logs through the mobile application. The presentation of results in this section is divided into four folds: (a) The feature engineering analysis results from both input data sources (i.e., HRV and textual logs); (b) The classification of HRV results; (c) The RNN text analysis and prediction results; (d) The decision fusion of both (b and c) results. Finally, we discuss the results and reflect on essential aspects of the features to deploy the model in subsequent studies.

### 4.1. Features Interpretation

**Figure 4:**
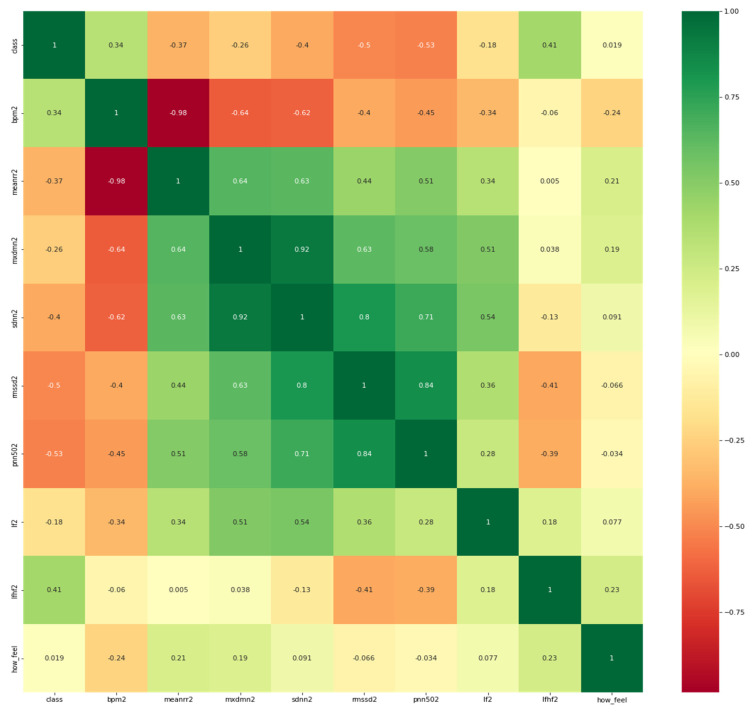
Heatmap of the features and the target variable (i.e., class).

To better understand the correlation of each HRV feature with the target which is potential COVID-19 infection or no evident signs of the infection, it is necessary to visualize the correlation map between them. It is worth mentioning that participants were asked to report their feelings in response to the question: “how do you feel?”as it appears in the heat map in Figure 4 below.

**Figure 5:**
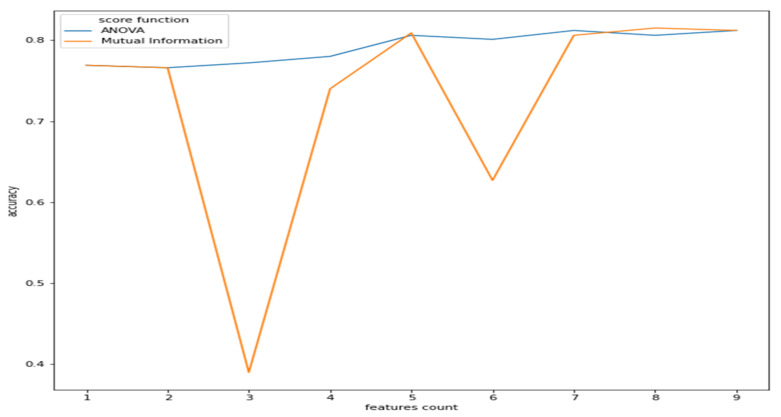
Features count vs. accuracy (ANOVA and MI).

As observed from Figure 2, the features have a reasonable correlation with the target value called “class”and with the question response how you feel? However, the goal is to correlate well between the features and the target and not between the features themselves. Therefore, we applied filter feature selection methods that assess the correlation of each feature with the target. ANOVA-F value and mutual information (MI) methods were applied. Figure 5 shows at which feature count the feature score stabilizes.

**Figure 6:**
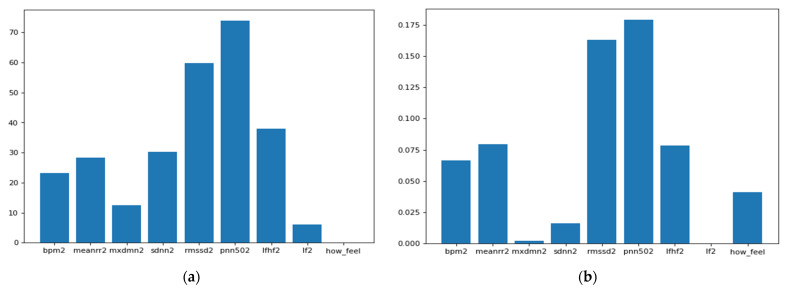
This Figure shows the features ranking according to their importance to the target through two different feature selection techniques which are: (**a**) ANOVA-F and (**b**) Mutual Information.

As we can see from Figure 5, the ANOVA-F value stabilizes faster than MI as features count. We observe that at five features, the accuracy of both methods is performing well. To better understand these features and their ranking, Figure 6a,b shows each feature score according to ANOVA-F value and MI methods, respectively. In Figure 6a, as ANOVA-F shows, pNN50, RMSSD, SDNN, and LF/HF rank the best among other features. Likewise, MI in Figure 6b shows that pNN50, RMSSD, LF/HF, and interestingly the question how you feel, which is a post-measurement survey. The response is a Likert scale from −2 (worse) to +2 (the best).

**Figure 7:**
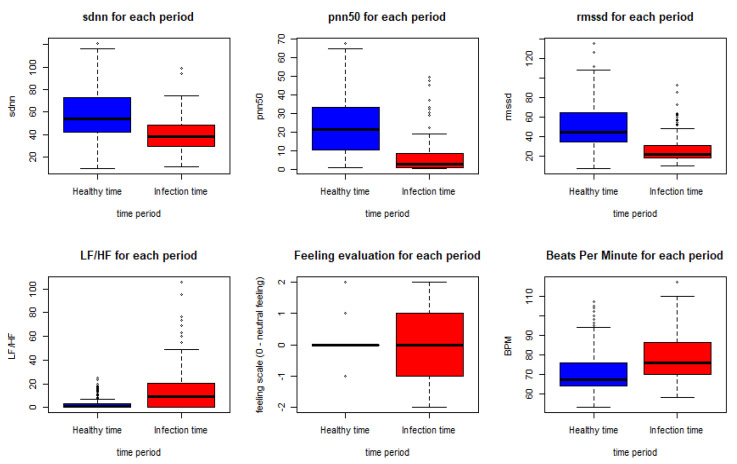
HRV features, BPM and feeling assessment distribution in the healthy and the infection time periods.

These results come in line with previous recent studies such as [32,33], which used RMSSD and other features to predict the illness of COVID-19 on a specific day which gives it the potential to represent an early biomarker of illness. Another recent study in [46] used HRV components to pre-empt affliction severity in a case study on SARS-Cov-2 symptoms. In [46], Tanwar et al. used features like RMSSD, SDNN, LF, and HF to create the internal states of the hidden Markov model (HMM) based on the presence and the absence of a consistent decline in these measures. In our model, we trained five main HRV components and one non-biometric feature (feeling self-assessment). The HRV features used to train the first classifier are shown in Figure 7 below.

As shown in the Figure below, using the Wilcoxon nonparametric test gave a *p*-value < 0.0005 for all the features except for the feeling assessment, which was 0.8931. Thus, we can conclude a significant statistical difference between the biometrics in the meantime periods of infection and the meantime periods before the infection.

**Figure 8:**
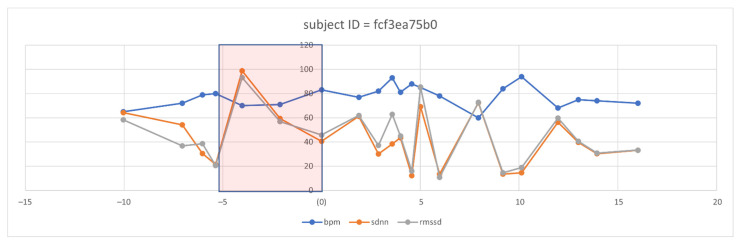
Observation of selected HRV features and BPM overtime in one subject before and after the symptom’s onset.

However, even with the promising results of the discriminant features of HRV, the need to visualize the features changes over time. Therefore, we selected three discriminant features, i.e., RMSSD, SDNN, and BPM (because it is inversely proportional in change with HRV features). We defined a five-day time window baseline before the symptoms and a range of five days after the onset of symptoms. After visualizing several patient cases, we could observe clear changes in some of these features, such as the following case that appears in Figure 8 for an individual.

From the five-day window, we can observe that two days ahead of the onset of symptoms, the mean of BPM tended to increase in successive times of the days above the baseline, which is then taken as the mean of the individual history of readings. Similarly, we can notice the successive decays in the SDNN and the RMSSD metrics before the onset of the symptom day (N = day 0). Interestingly, we observed this pattern repeated itself before day 10. We analyzed the user logs and survey answers on the 10th day and five days after, noticing that the subject expressed excessive illness for the first time since the onset of the symptoms. We are aware that this case does not motivate us to generalize to a large diverse population. However, observing the signal behavior in response to the COVID-19 onsets previously known and recorded is vital.

### 4.2. Heart Signals and Feelings Classification Results and Their Interpretations

After visualizing the features and testing them statistically, we trained the best-selected features with different state-of-the-art classifiers as appears below in Table 2. Here in this Table, we can see how the model can distinguish between healthy and non-healthy measurements and even responses. We can notice different modalities. Specifically, HRV features fused with the “how do you feel” response feature. SVM scores the best performance in terms of accuracy, precision, and recall. The second modality is considering the HRV features only. We can notice how the performance dropped in all the classifiers except the logistics regression. Finally, the modality with just the reported response of “how do you feel” is considered the worst in terms of performance. This is clear since the response cannot represent inflammation promptly nor specific to the COVID-19 illness.

**Figure 9:**
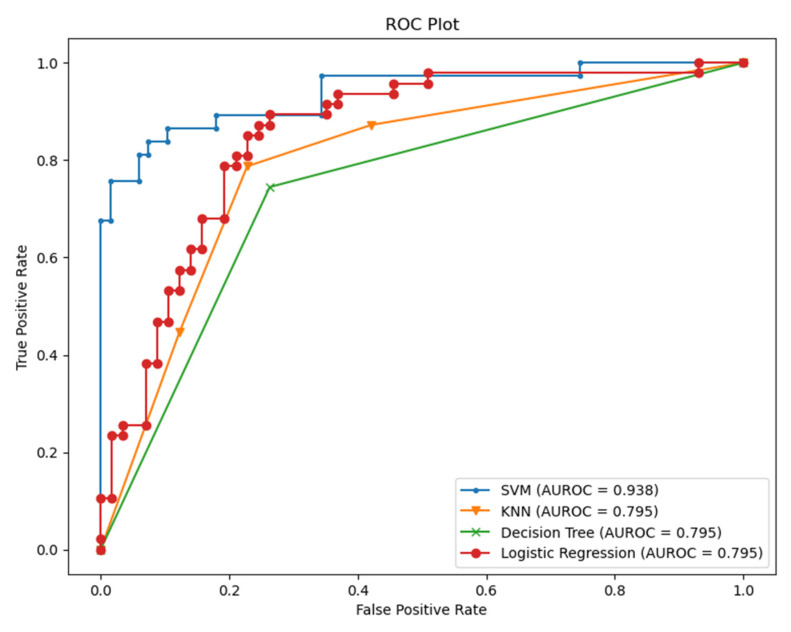
AUC-ROC for the classifiers (HRV + Feeling features).

From Table 2, we conclude that fusing the classifier with relevant information about the context helps to improve the performance of the classifiers. HRV alone could be misleading in various situations. HRV can be sensitive to emotions, cardiovascular disorders, and other illness responses. However, it is still a very promising measure, especially when it comes to assessing the severity, the acute response of the COVID-19, and estimating the probability of hospitalization needed for those patients [11]. Therefore, we checked the specificity and the sensitivity of the model by visualizing the area under the receiver operating characteristic curve (AUC-ROC) for the modality of HRV and feeling assessment, as shown in Figure 9.

**Figure 10:**
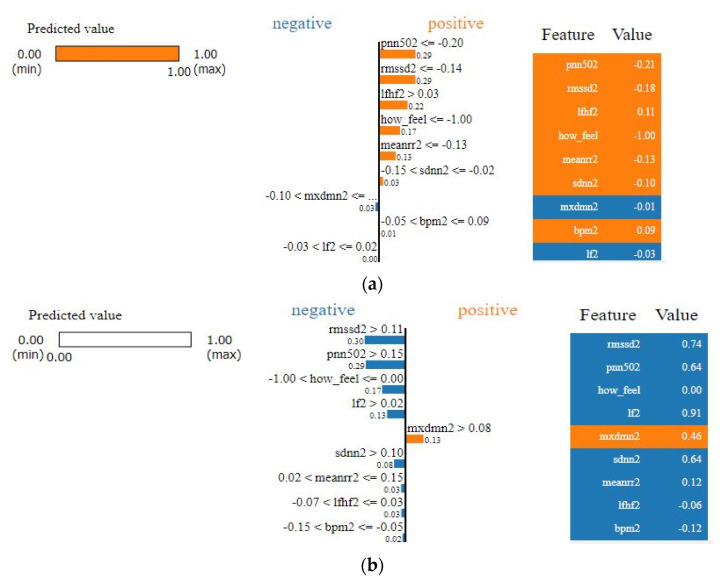
LIME explanation of the SVM classifier (**a**) for a positive case; (**b**) for a negative case.

As we see from Figure 9, it is apparent that SVM surpasses other classifiers in terms of sensitivity and specificity with an AUC value of 0.938. The least performer appears to be the decision tree where it scored AUC of 0.795. However, the decision tree has an advantage over other types of classifiers, which is interpretability (i.e., explainability). This is important since decisions related to human well-being could not be taken by a grant from an AI black-box model. Therefore, we selected the SVM classifier and added a new layer on the top of the classifier which is a decision explainer. The decision explainer is simply a local interpretable model-agonistic explanation (LIME) [47]. LIME attempts to modify individual data samples and observe the impact on the model output. This mimics the human curiosity in knowing why was this prediction made and which variable affected the decision? The explainer output should reflect the contribution of each feature to the prediction of each data sample (i.e., local interpretability). LIME defines an explanation as a model g∈G, G represents the potential explainable models, such as linear models, decision trees, or rule lists. These models should readily demonstrate visual or textual explanations such as the visual representation given in Figure 10. Figure 10 shows two examples of LIME explanations applied on SVM (because it outperformed other classifiers). Figure 10a shows a potentially COVID-19 case (i.e., positive prediction: 1).

On the right-hand side of Figure 10, each feature is given a weight based on its importance to the target. In LIME taxonomy [47], this is calculated by re-sampling fake data points perturbing the real observation data (i.e., locality) and then computing the distance between the generated point and the observation. In our context, we can see that pNN50, RMSSD, LF/HF, how feel, and meanrr are the top five important features. To the left of the names and scores of the features in Figure 10a, the LIME explains why the decision was positive. For example, we see the pNN50 fell below the threshold (i.e., −0.20) and it is −0.21. Thus, it is classified as “positive” which means potentially having COVID-19. Other features like RMSSD also dropped below the threshold, which is −0.14, by 0.4 points, whereas LF/HF increased above the threshold, which is 0.03, by 0.08 points. Interestingly, BPM was on the boundary of the decision but witnessed to increase slightly above it. Finally, SDNN dropped 0.08 points. These interpretations are in line with the established correlations between HRV features and COVID-19 symptoms [22,48]. In [48] for example, it clearly shows that COVID-19 patients had a significantly lower SDNN (*p* < 0.001) and a higher LF/HF (*p* = 0.016). Likewise, in Figure 10b the negative decision (i.e., no sufficient evidence of COVID-19 infection) can be easily interpreted by opposing the correlations mentioned in the positive case (i.e., Figure 10a). LIME showed a high degree of interpretability [47], which could imply that the results of our model can be comprehended by non-experts, which is a necessity in medical applications.

### 4.3. Daily Textual Logs Classification Results

In our framework, we used the AWD-LSTM (asynchronous stochastic gradient descent (SGD) weight-dropped LSTM). The baseline of this model consisted of (a) Embedding, which is a default Pytorch layer to store and retrieve word embeddings, (b) the DropOut to avoid overfitting by disabling the neurons equal to 30%, (c) LSTM to extract words dependencies and information, and (d) classifier layer to classify between log texts that represent a status of a COVID-19 patient or a healthy person. We pretrained our model using the universal language model fine-tuning (ULMfit) trained on wikitext and fine-tuned on our logs data from the users while using the Welltory application. The performance results are presented in Table 3 below.

**Figure 11:**
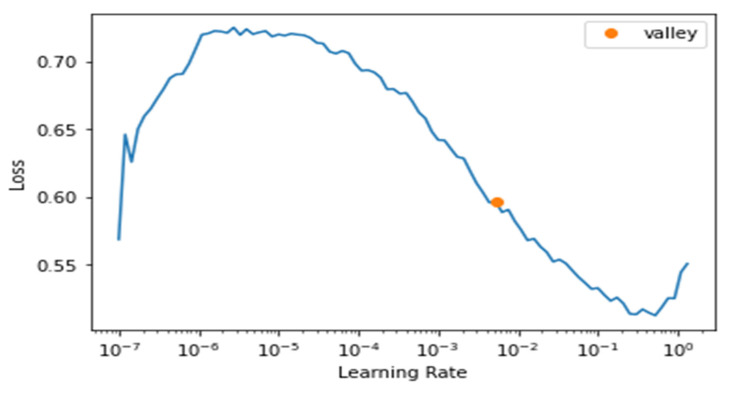
Learning Rate and Loss of fastai.

We maintained monitoring of the learning rate to choose the best parameters for the model, as we see from Figure 11 below that the best learning rate achievable was around 10^−2^.

After that, we tested the model with different statements like (“I feel tired and need some rest”). The model was able to classify different sentences as “potentially COVID-19 infected” or “no clear evidence of infection.” Each classification is provided with the probability P of the outcome. For example, the last sentence was classified as “potentially COVID-19 infected” with a probability of 81.49%. The final decision would comprise linearly adding this probability-weighted with 0.3 and the probability of the HRV classification over a time course of 2 days. If the two classifiers have higher probabilities of the infection, an alert comes to a screen showing abnormal changes in the measured data.

## 5. Threats to Validity

First, this study used a publicly available dataset that was relatively limited in terms of participants and the history recorded of ‘patients’ data. This is a limitation that challenged us to establish a physiological signals baseline. However, the use of this dataset enabled us to evaluate the approach and attempt to prove that those measures with other fused supporting features could be used as an early detection alarming for the infection. The next step will be generating our dataset and recruiting as many volunteers as possible to have more confident data and more statistical power.

Second, we were not able to contact the participants to ask them firmly about their reported subjective assessment of symptoms, such as fatigue and headache, to be able to verify those symptoms were just specific to the COVID-19 infection or not.

Third, the inconsistent readings of the heart rate for some patients made us exclude some records of data that affected the overall performance.

Fourth, although our language model using NLP showed successful performance in classifying between textual health statuses of participants, the threat remains because we do not assume all participants to express the same way when they feel unwell. Moreover, for asymptomatic patients, this addition will be almost useless.

Finally, we are aware that the variations in HRV signals are not specific to COVID-19 infection, and thus we took this into account when fusing the multimodal with other classifiers results, such as the early feeling and symptoms classifier. Furthermore, the output of this model would be an alarming message and not a test result of the COVID-19.

## 6. Conclusions and Future Work

This paper aimed at investigating the viability of using physiological signals such as HRV acquired from wearable devices (e.g., smartwatches and bracelets) over time and other symptoms classification to detect COVID-19 infection onset at least two days before the onset of tough symptoms. The physiological signals, including the HRV measures, were collected from users through a mobile application and wearable devices over a continuous period. The signals were then statistically analyzed to extract and select the most discriminating features. The features were chosen to be specific and sensitive to identify infection patterns, including the COVID-19 ones. The features selection methods and the exploratory data analysis showed the importance of some HRV time-domain features, and the frequency domain features relative to the target value (i.e., potential infection vs. no evidence). Examples of the important features included RMSSD, SDNN, pNN50, and LF/HF, which is in line with recent studies addressing the same topic. We found that fusing the HRV features with the feeling self-assessment measures can yield better performance. Therefore, we enlarged the window of the contextual information and used NLP fastai library to analyze the daily textual logs entered by participants. The work resulted in an accuracy of 83.34 ± 1.68% with 0.91, 0.88, and 0.89 precision, recall, and F-Score, respectively, in predicting the infection two days before the onset of the symptoms. The LIME framework introduced to explain the results showed a better interpretation of each feature and its contribution to the final decision. From another angle, we could notice that analyzing the daily textual logs of users could be a successful approach in detecting “special” patterns of text written by patients before they got infected with acceptable accuracy. People tended to write observable types of logs before the onset of the symptoms. Nonetheless, we are still conservative about the viability of analyzing ‘participants’ textual logs since nothing will be of significant value in the asymptomatic patients. The research outcome is expected to significantly help prevent the spread of disease, take the right action, and monitor the status of the patient remotely. As for future work, we are proposing a multimodal fused model of biofeedback wearable non-intrusive sensors (e.g., heart rate variability (HRV)) and recorded cough sound signals acquired through smart mobile phones to predict the onset of COVID-19 symptoms using manifestations of the autonomic nervous system (ANS), such as the modulation of heartbeats affected by the immunity system response to the infection and the sound analysis of the cough.

Our next paper will be based on providing a mobile application that is synced with wearable devices, collecting consistent and continuous data from volunteers after taking all ethical permissions required by the countries in which the system will be used. The data analysis will depend on many sophisticated models and techniques, such as deep learning and neural networks.

## Figures and Tables

**Table 1:** Dataset Description.

Source	Data	Details
Welltory Mobile Application	HRV	Daily readings of beats per minute and HRV features such as SDNN, RMSSD, pNN50, COVID-19 onset date. Moreover, textual tags were provided by patients about their status daily.
	Blood pressure	Diastolic and systolic readings, functional change index.
	Heart Rate	Beats per minute readings, and a binary answer (whether heart rate was measured at rest).
	Surveys	COVID symptoms such as cough assessment, fever, breath shortness, fatigue, etc.
Wearables	Physiological metrics and fitness data	Resting heart rate, heart rate, oxygen saturation, steps count, walking distances.
	Sleep data	Sleep begins and ends, sleep duration, light, and deep sleep information.

**Table 2:** Heart signals prediction results and performance—time window = 2.

Modality	Model	Accuracy	Precision	Recall	F1-Score
HRV features + feeling assessment	SVM	83.34 ± 1.68%	0.91	0.88	0.89
	KNN	83.06 ± 1.99%	0.80	0.80	0.80
	Decision Tree	74.28 ± 0.613%	0.80	0.79	0.79
	Logistic Regression	78.93 ± 2.34%	0.80	0.80	0.79
HRV features only	SVM	78.85 ± 3.04%	0.79	0.81	0.78
	KNN	80.17 ± 0.28%	0.75	0.75	0.75
	Decision Tree	76.30 ± 0.98%	0.71	0.71	0.71
	Logistic Regression	79.75 ± 4.31%	0.79	0.79	0.79
Feeling assessment only	SVM	65.38 ± 8.21%	0.66	0.67	0.65
	KNN	41.74 ± 9.68%	0.46	0.47	0.41
	Decision Tree	50.58 ± 7.03%	0.57	0.55	0.53
	Logistic Regression	58.68 ± 11.28%	0.27	0.50	0.35

**Table 3:** Performance results of the fastai LSTM model.

Epoch	Train Loss	Valid Loss	Accuracy
0	0.362183	0.264590	0.925373
1	0.355476	1.288716	0.686567
2	0.461005	0.675994	0.791045
3	0.447026	0.774474	0.805970
4	0.420781	0.499611	0.805970

## Data Availability

The data is available at: https://github.com/Welltory/hrv-covid19 (accessed on 10 November 2021).
